# Sequential Atraumatic Insufficiency Fractures of the Distal Fibula and Tibia: A Case Report

**DOI:** 10.7759/cureus.117268

**Published:** 2026-09-30

**Authors:** Fran Shing Fung Kwok, Chanaka Nissanka, Barry Ka Yu Chan

**Affiliations:** 1 Radiology, Pamela Youde Nethersole Eastern Hospital, Hong Kong, HKG; 2 Radiology, District General Hospital, Matara, LKA

**Keywords:** biomechanics, distal fibula, distal tibia, fragility fracture, insufficiency fracture, osteopenia, postmenopausal women, stress fracture

## Abstract

Insufficiency fractures result from physiological loading of bone with reduced mechanical resistance and may occur at uncommon sites in individuals with underlying skeletal fragility. Distal fibular insufficiency fractures are uncommon, and sequential involvement of the ipsilateral distal tibia appears particularly unusual. We report a 73-year-old postmenopausal woman who presented with atraumatic left ankle pain and swelling and was found to have a distal fibular fracture. Approximately two months later, while walking on level ground, she developed new lower-leg pain and swelling without trauma. Radiographs demonstrated interval healing of the fibular fracture together with a new nondisplaced distal tibial fracture. Subsequent computed tomography (CT) demonstrated a diffuse osteopenic appearance of the visualized bones, progressive healing of both fractures, and no focal or infiltrative osseous lesion. Both fractures healed progressively with non-operative management, and subsequent bone-health assessment demonstrated osteopenia. A focused narrative review of the literature identified isolated, bilateral, and recurrent distal fibular stress fractures, as well as concurrent distal tibial and fibular fractures, but no directly comparable distal fibula-to-ipsilateral-distal-tibia sequence. This case highlights the potential importance of an initial insufficiency fracture as a sentinel fragility event. New or changing symptoms after such an event should prompt consideration of an additional insufficiency fracture, together with assessment of systemic skeletal health and potentially contributory biomechanical factors, including altered loading during fracture recovery.

## Introduction

Low bone mineral density (BMD) represents a substantial and increasing global health burden, accounting for an estimated 17.2 million disability-adjusted life-years and 477,000 deaths worldwide in 2020 [1]. Low bone mass is also common locally; in a Hong Kong screening cohort of 1,800 women aged 65 years or older, 60.4% had osteopenia and 23.7% had osteoporosis on dual-energy X-ray absorptiometry (DXA) assessment [2]. In older adults, reduced BMD may be associated with age- and menopause-related bone loss, low body mass index (BMI), reduced physical activity, nutritional deficiency, and medications affecting bone metabolism [2].

Stress fractures are conventionally divided into fatigue fractures, resulting from abnormal repetitive stress applied to otherwise normal bone, and insufficiency fractures, resulting from physiological stress applied to bone with reduced mechanical resistance [3]. BMD is commonly assessed using DXA; in postmenopausal women, a T-score between −1.0 and −2.5 indicates osteopenia, whereas a T-score of −2.5 or lower is diagnostic of osteoporosis [2]. The absence of an identifiable traumatic event in an older patient with a fracture should therefore raise suspicion for an insufficiency fracture and underlying skeletal fragility. Although insufficiency fractures are well recognized in the lower extremity, involvement of the distal fibula is rare, with the published literature consisting predominantly of individual reports and small series [4-11]. Reported predisposing factors encompass both impaired bone strength and abnormal mechanical loading [4-11]. Management generally combines appropriate fracture protection and rehabilitation with assessment and treatment of underlying skeletal fragility and modifiable risk factors [12].

Importantly, an initial fragility fracture may represent a sentinel event for subsequent fracture. Contemporary osteoporosis guidance specifically uses this concept, while systematic-review and large cohort data demonstrate that fracture risk is greatest soon after an initial fracture and remains elevated beyond that explained by BMD alone [12-14]. This report describes an atraumatic distal fibular insufficiency fracture in a postmenopausal woman followed approximately two months later by a new ipsilateral distal tibial insufficiency fracture. This sequential presentation highlights the potential importance of an initial insufficiency fracture as a clinical warning of continued fracture susceptibility.

## Case presentation

A 73-year-old postmenopausal woman presented with a history of left ankle pain and swelling, with no preceding trauma. Her symptoms had initially been treated as gout. Her medical history included gout, diabetes mellitus, hypertension, hyperlipidaemia, and chronic kidney disease; the mean estimated glomerular filtration rate (eGFR) was 34.7 mL/min/1.73 m² across five measurements between July 2025 and July 2026. Serum urate was 0.34 mmol/L. Pertinent bone and mineral biochemistry was within reference ranges, including adjusted calcium 2.33 mmol/L, phosphate 1.10 mmol/L and alkaline phosphatase (ALP) 120 U/L in June 2026, and parathyroid hormone (PTH) 5.2 pmol/L in October 2025. Persistent symptoms subsequently prompted further assessment.

Radiographs obtained in July 2025 demonstrated a transverse fracture of the distal left fibular shaft (Figure 1). At orthopaedic review, she was walking independently and had mild focal tenderness over the distal fibula without overlying skin abnormality or palpable mass. The fracture was managed non-operatively.

**Figure 1:**
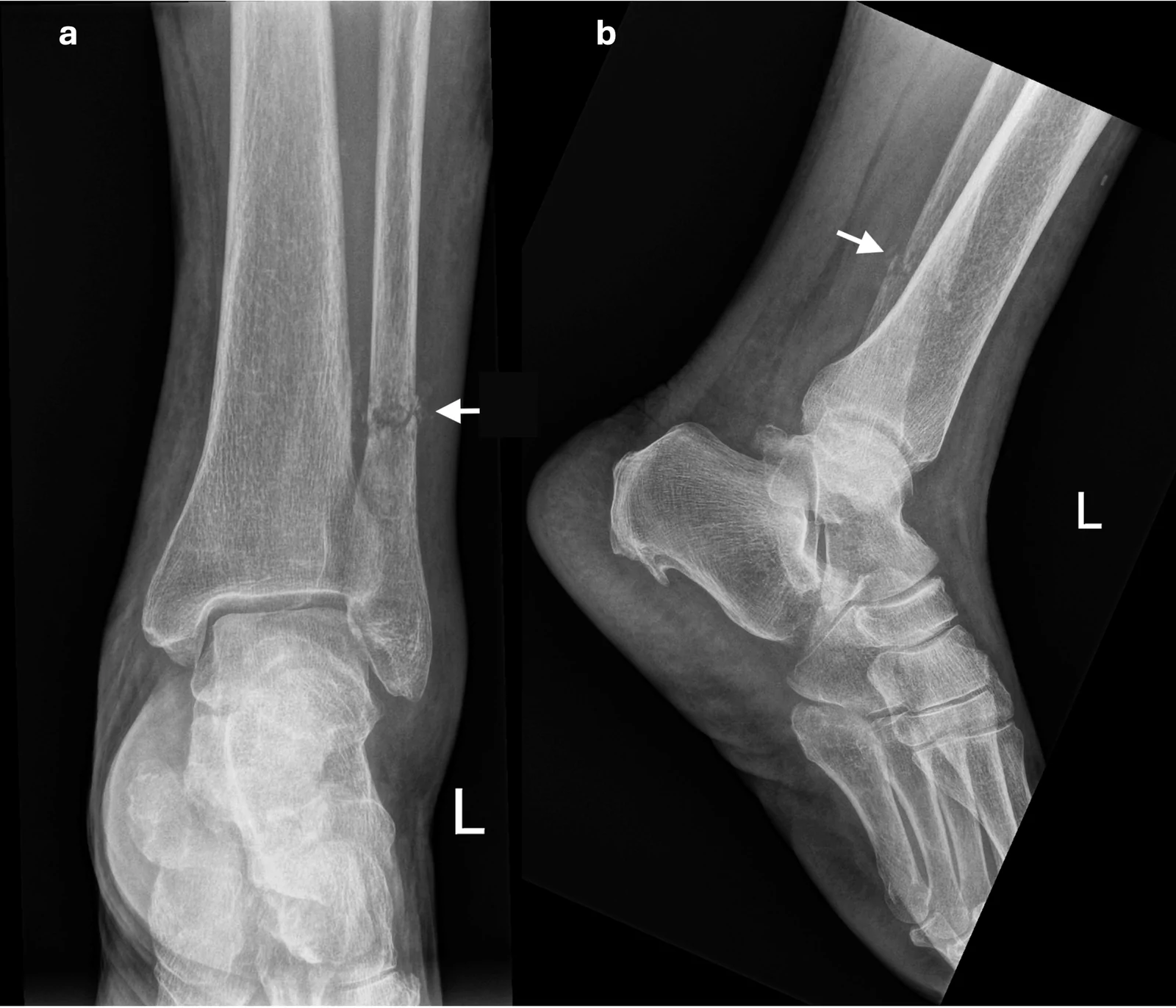
Initial distal fibular fracture. Anteroposterior (A) and lateral (B) radiographs of the left ankle obtained at initial presentation demonstrate a transverse distal fibular shaft fracture (straight arrows). No distal tibial fracture is present.

Approximately two months later, while walking on level ground with her ankle brace, she developed sudden worsening left lower-leg pain and swelling while weight-bearing. There was again no traumatic event. Radiographs demonstrated the previously identified distal fibular fracture with interval healing and mild angulation without significant displacement, together with a new nondisplaced transverse distal tibial fracture (Figure 2).

**Figure 2:**
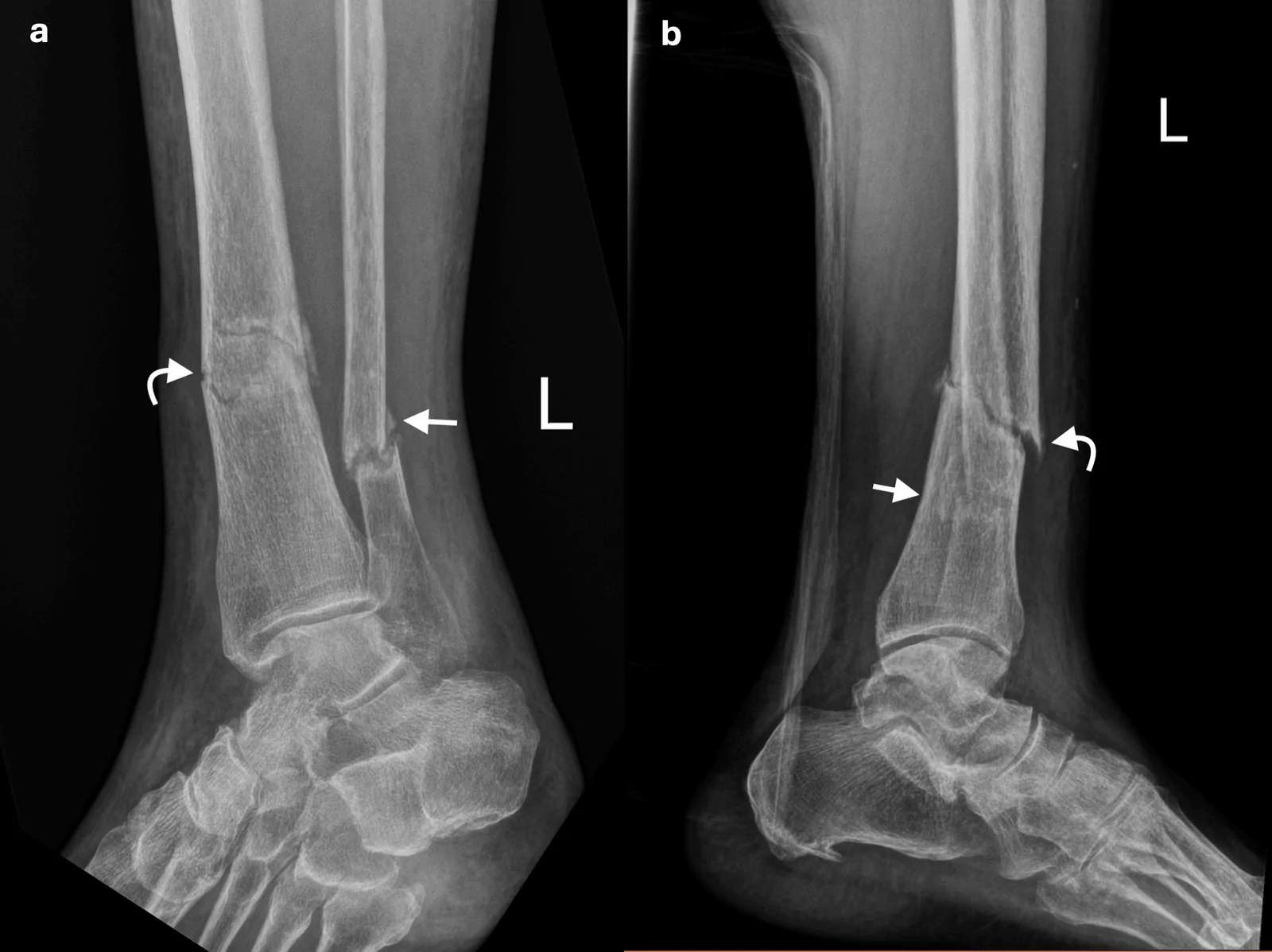
Subsequent distal tibial fracture. Anteroposterior (A) and lateral (B) radiographs obtained approximately two months later demonstrate interval healing of the transverse distal fibular fracture (straight arrows), with mild angulation and no significant displacement, together with a new nondisplaced transverse distal tibial fracture (curved arrows).

Computed tomography (CT) performed approximately one month later demonstrated a diffuse osteopenic appearance of the visualized bones, with progressive healing of both fractures. There was relatively advanced healing of the distal fibular fracture and developing healing of the distal tibial fracture, without a focal or infiltrative osseous lesion (Figure 3A). Taken together with the atraumatic clinical presentation and imaging appearances, the findings were considered compatible with sequential insufficiency fractures. The patient was managed non-operatively with immobilization and protected weight-bearing, followed by progressive mobilization. Serial radiographs demonstrated increasing callus formation with maintained alignment. By approximately four months after the tibial fracture, she was fully weight-bearing without pain. Follow-up CT approximately six months after the second fracture demonstrated complete union of the distal fibular fracture and near-complete union of the distal tibial fracture (Figure 3B). Subsequent radiographs showed continued healing, and by nine months she was independently ambulant and asymptomatic.

**Figure 3:**
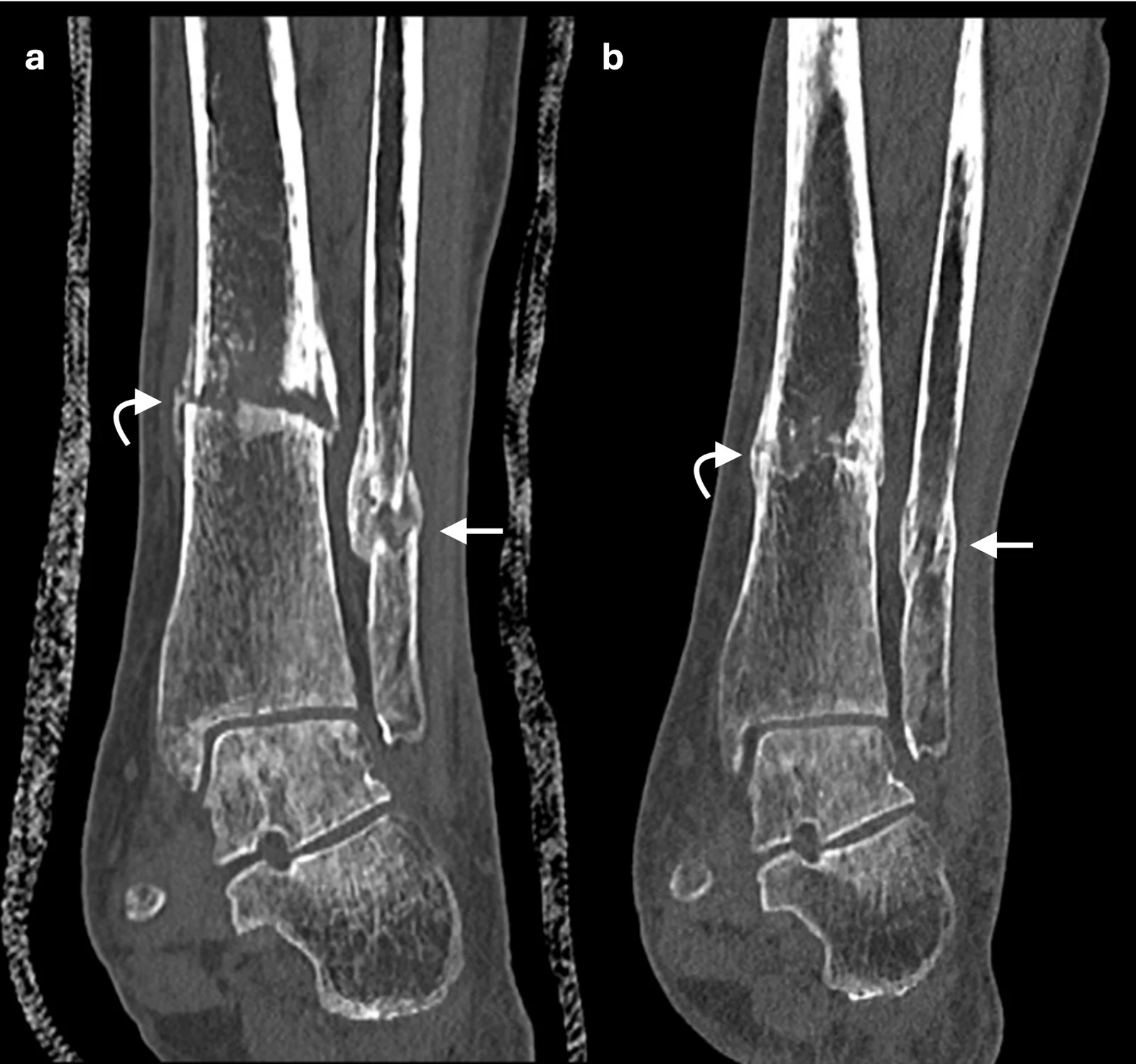
Computed tomography evolution of fracture healing. Coronal reformatted CT image obtained approximately one month after the distal tibial fracture (A) demonstrates relatively advanced healing of the distal fibular fracture (straight arrow) and developing healing of the distal tibial fracture (curved arrow). A follow-up coronal reformatted CT image obtained approximately six months after the distal tibial fracture (B) demonstrates complete union of the distal fibular fracture (straight arrow) and near-complete union of the distal tibial fracture (curved arrow).

Bone-health assessment subsequently demonstrated osteopenia, with a hip T-score of −2.0 and lumbar spine T-score of −1.1. Her calculated 10-year fracture probabilities were 19% for a major osteoporotic fracture and 7% for hip fracture. Pharmacological bone-protective treatment was discussed, but treatment was deferred while planned dental work was being completed.

## Discussion

Insufficiency fractures occur when physiological loading is applied to bone with reduced mechanical resistance, in contrast to fatigue fractures, which result from abnormal repetitive loading of otherwise normal bone [3]. Although insufficiency fractures are well recognized in older adults, distal fibular involvement appears uncommon. Published reports consist largely of individual cases and small series, with presentations including isolated distal fibular fractures, bilateral or recurrent fibular fractures, and concurrent distal tibial and fibular fractures [4-11] (Table 1). A focused search of PubMed and relevant reference lists was undertaken for reports of distal fibular stress and insufficiency fractures; this was intended as a focused narrative review rather than a systematic review. No directly comparable report of an atraumatic distal fibular insufficiency fracture followed by an ipsilateral distal tibial insufficiency fracture was identified.

**Table 1:** Focused literature review of adult distal fibular stress and insufficiency fractures BMD: bone mineral density; DEXA: dual-energy X-ray absorptiometry; THA: total hip arthroplasty.

Author	Sex; Age	Fracture location and pattern	Bone health/predisposing factors
Kaye [4]	11 postmenopausal women; mean age 62 years (42-83)	Twelve foot/ankle insufficiency stress fractures, including 3 distal fibular fractures.	Cohort-level factors: all postmenopausal; radiographic osteoporosis in 4/11; prior fracture in 4/11 and possible previous stress fracture in 2/11. Reported osteoporosis-associated factors included smoking, thiazide diuretic use, soft-drink consumption, eating disorder, and intestinal bypass. 6 of 11 had a foot deformity. These factors were not specifically attributable to the distal-fibular cases.
Kazimoglu et al. [5]	1 woman; age 54 years	Bilateral distal fibular stress fractures; left followed by right.	Osteoporosis (spinal T-score -2.6; femoral T-score -1.3); postmenopausal; recent high-tempo weight-bearing walking.
Mpofu et al. [6]	1 woman; age 45 years	Simultaneous bilateral distal tibial and fibular stress fractures.	Normal BMD and metabolic investigations; 25 pack-year smoking history, bilateral ankle valgus and surgically induced amenorrhoea discussed as potential contributors.
Cheng et al. [7]	1 woman; age 44 years	Isolated left distal fibular stress fracture, approximately 3 cm proximal to the lateral malleolus.	Stage III rigid flatfoot with fixed hindfoot valgus; repetitive occupational walking/overtime. Routine laboratory investigations normal; BMD not reported.
Kojima et al. [8]	1 woman; age 54 years	Distal tibial and distal fibular insufficiency fractures identified at the same presentation, 22 weeks after total hip arthroplasty.	Lumbar T-score -2.5; generalized and local disuse osteoporosis; increased postoperative loading and altered mechanics proposed.
Liu et al. [9]	6 women; mean age 58 years (range 45–64)	Six distal fibular stress fractures; mean fracture level 5.8 cm (4.2-7 cm) proximal to the lateral malleolar tip. One recurrent fracture reported.	Planovalgus deformity with hindfoot valgus in all six; 4/6 had osteopenia and/or osteoporosis by DEXA.
Srinivasan et al. [10]	1 woman; age 69 years	Distal fibular insufficiency fracture associated with advanced planovalgus deformity.	Osteopenia; severe planovalgus/advanced tibialis posterior dysfunction with altered fibular loading.
Nishino et al. [11]	1 woman; age 67 years	Isolated transverse distal fibular stress fracture, 5 cm proximal to the lateral malleolar tip, 5 months after total hip arthroplasty.	Osteopenic-range DEXA; vitamin D deficiency; postoperative valgus alignment change and possible local disuse osteoporosis.

The clinical presentation may be deceptively nonspecific. In the present case, ankle pain and swelling had initially been treated as gout. Similarly, Mpofu et al. described bilateral ankle swelling that was initially managed as peripheral oedema with diuretic therapy before the underlying fractures were recognized [6]. These examples illustrate how distal lower-leg and ankle swelling may initially be attributed to more common inflammatory or systemic causes. Persistent focal pain, tenderness, or otherwise unexplained swelling should therefore prompt consideration of an underlying stress or insufficiency fracture, particularly in the presence of skeletal fragility or abnormal lower-limb biomechanics.

The sequential occurrence of the two fractures is particularly relevant in the context of subsequent-fracture risk. A previous fragility fracture is an established marker of increased risk of further fracture, with the excess risk being greatest soon after the initial event [12-14]. Contemporary osteoporosis guidance has accordingly described an initial fracture in an older adult as a sentinel event, identifying a period of heightened fracture susceptibility [12]. In the present case, the distal fibular fracture should not be interpreted as having directly caused the subsequent tibial fracture; rather, the two fractures may represent sequential manifestations of persistent underlying susceptibility to insufficiency injury, potentially influenced by both skeletal fragility and altered mechanical loading. New or changing symptoms following an initial fracture should therefore not automatically be attributed to the known injury, and an additional insufficiency fracture should remain within the differentials.

The published distal fibular literature also highlights the potential contribution of local biomechanics. Cheng et al. reported a distal fibular stress fracture associated with rigid flatfoot and hindfoot valgus [7], while Liu et al. identified planovalgus deformity with hindfoot valgus in all six patients in their series [9]. Srinivasan et al. similarly attributed fibular insufficiency fracture to altered loading associated with advanced tibialis posterior dysfunction [10]. Altered lower-limb alignment or loading following arthroplasty was also implicated by Kojima et al. and Nishino et al. [8,11]. Collectively, these reports suggest that distal fibular stress fractures may reflect not only impaired bone strength but also redistribution of mechanical load through the lower limb. In addition to these relatively fixed structural factors, transient alterations in loading during fracture healing may also be relevant. The initial fibular fracture, together with immobilization and altered gait during recovery, may temporarily redistribute mechanical stress across the lower leg and potentially increase tibial loading, although this mechanism cannot be established retrospectively in the present case.

This has potential clinical relevance because some mechanical contributors may be amenable to modification. Following an atraumatic insufficiency fracture, assessment may therefore reasonably extend beyond BMD to include consideration of lower-limb alignment, hindfoot valgus, planovalgus deformity, gait alteration, and other sources of abnormal loading where clinically appropriate. Attention to gait and loading mechanics during rehabilitation, with preservation of the patient’s usual gait and loading pattern as far as clinically appropriate, may also be relevant. Weight-bearing alignment radiographs were not obtained because loading was minimized in the setting of acute symptomatic fractures; consequently, potential biomechanical contributors could not be formally assessed in this patient. Furthermore, the existing literature does not establish that correction of these abnormalities reduces subsequent insufficiency-fracture risk. Whether identification and treatment of potentially modifiable biomechanical factors after an initial insufficiency fracture can reduce subsequent fracture susceptibility represents a potential area for future investigation.

The subsequent bone-health assessment in this patient demonstrated osteopenia rather than densitometric osteoporosis. This does not exclude clinically important skeletal fragility. Large cohort data have demonstrated that substantial numbers of fragility fractures occur in individuals with osteopenic-range BMD, while a previous fracture confers increased subsequent fracture risk beyond that explained by BMD alone [14,15]. The fracture history therefore remains clinically relevant even when the T-score does not meet the conventional densitometric threshold for osteoporosis.

The focused literature review identified isolated and bilateral distal fibular fractures, simultaneous bilateral distal tibial and fibular fractures, and distal tibial and fibular fractures identified concurrently following arthroplasty. A sequential pattern involving the proximal fibula followed by the proximal tibia has also been reported by Cheung et al. [16], providing a precedent for sequential insufficiency fractures involving the two bones at a different anatomical level. However, no directly comparable distal fibula-to-ipsilateral-distal-tibia sequence was identified. The present case therefore supports considering an atraumatic distal fibular insufficiency fracture as a potential sentinel fragility event: subsequent new or changing lower-leg symptoms should prompt reassessment for an additional insufficiency fracture, together with evaluation of systemic skeletal health and, where appropriate, potentially contributory biomechanical factors.

## Conclusions

Sequential atraumatic insufficiency fractures of the distal fibula and ipsilateral distal tibia are uncommon. An initial distal fibular insufficiency fracture may represent a sentinel fragility event and should heighten clinical vigilance when new or changing lower-leg symptoms develop. Such symptoms should prompt consideration of an additional insufficiency fracture rather than being attributed automatically to the known injury. Assessment of systemic skeletal health and, where clinically appropriate, potentially modifiable biomechanical contributors, including both fixed structural factors and transient alterations in loading during fracture recovery, may also be valuable, although whether correction of these factors reduces subsequent fracture risk requires further investigation.
